# Clinical analysis of small cell carcinoma of the bladder in Chinese: nine case reports and literature reviews

**DOI:** 10.1186/s12957-016-1079-y

**Published:** 2017-01-26

**Authors:** Zhi Chen, Qingquan Liu, Ruibao Chen, Zhuo Liu, Mingchao Li, Qing Ling, Licheng Wu, Jun Yang, Xiaming Liu, Tao Wang, Zhiquan Hu, Xiaoling Guo, Shaogang Wang, Weiming Yang, Jihong Liu

**Affiliations:** 1 Department of Urology, Huazhong University of Science and Technology, Wuhan, 430030 China; 2 Institute of Urology, Tongji Hospital, Tongji Medical College, Huazhong University of Science and Technology, Wuhan, 430030 China; 3 Department of Geriatrics, Tongji Hospital, Tongji Medical College, Huazhong University of Science and Technology, Wuhan, 430030 China

**Keywords:** Bladder cancer, Small cell carcinoma, Neuroendocrine carcinoma, Chemotherapy, Radiotherapy

## Abstract

**Background:**

Small cell carcinoma of the bladder (SCCB) is a kind of rare and highly aggressive tumor that is present in an advanced stage and has a propensity for early metastasis. The main presenting symptom of SCCB is hematuria. Surgery, chemotherapy, and radiotherapy, either alone or as ﻿a﻿ part of combined therapy, have been used as the treatment. The aim of this study is to present our experience with 9 SCCB patients who were treated with different modalities and to share the findings upon reviewing the literatures for patients with SCCB reported in 56 literatures in Chinese.

**Methods:**

We retrospectively evaluated 9 patients with SCCB from February 1980 to January 2014 in Tongji Hospital, Huazhong University of Science and Technology. The general characteristics, clinical manifestations, the pathological and immunohistochemical characteristics, treatment options, and prognostication in those eligible manuscripts were analyzed. In order to gain a better understanding of the clinical features of SCCB, another 119 cases reported in 56 articles were reviewed together (from January 1979 to March 2014). And a retrospective analysis was performed.

**Results:**

All the 9 cases in Tongji Hospital were successfully operated, and the tissue samples were sent for pathological examination. All the tumor tissues contained small cell carcinoma components. 4 cases coexisted with other histologic types of bladder cancers, and 2 out of the 9 cases had three different cell components. All the patients had muscle invasion, and 4 cases showed lymph nodes metastasis, 3 cases showed invasion of neighboring structures (seminal vesicle or uterus), and 1 case was highly suspected of liver metastasis. Immunohistochemistry results showed that PCK, Syn, NSE, and CD56 were all positive, but LCA was negative. After operations, 3 patients underwent chemotherapy and only 1 patient received postoperative radiotherapy. Patients were followed up, ranging from 3 to 84 months and the median survival time was 33 months. The leading cause of death was tumor recurrence or metastasis, while 2 patients are still alive. According to the published literature, the pathological stage, immunohistochemical markers, and survival curves of all the 128 cases were also retrospectively analyzed.

**Conclusions:**

SCCB is different from transitional cell carcinoma (TCC) of the bladder. It has its unique cytology, immunohistochemistry, and ultrastructural features. Its diagnosis relies on pathological examination and immunohistochemistry. The current main treatment for SCCB is surgery combined with chemotherapy. Since the disease develops early metastasis easily, the overall prognosis of this cancer is poor. Further research need to clarify the molecular pathogenesis so that novel therapies can be developed for this rare cancer.

## Background

Small cell carcinoma (SCC) is one subtype of the histopathological classifications with invasive biological behavior. It was mostly found in lung cancer. Small cell carcinoma outside the lungs only accounted for 4% of all small cell carcinomas. Small cell carcinoma of the bladder (SCCB) is very rare. Since first reported in 1981, its incidence accounts for about 0.5 ~ 1% of primary bladder tumor; there has been about 1000 cases reported so far [1]. The definite etiology and pathogenesis are not clear yet. Clinical treatments of SCCB have neither consistent correlation scheme nor a randomized clinical trial. Therapy modes include surgery, chemotherapy, and radiotherapy. Because of the rarity of this tumor, no prospective trials have been done to evaluate the optimal treatments [2]. The aim of the present study was to describe the clinical pathological features and outcomes of the nine patients in Tongji Hospital and to review the literatures.

## Methods

### Clinicopathological features

For this retrospective, observational study, the medical records data of the nine SCCB patients were obtained from Tongji Hospital, Huazhong University of Science and Technology from February 1980 to January 2014. The first case was diagnosed in March 1980 and the last in October 2013. Follow-up ended in January 2014. Clinical and demographic information obtained through the hospital records included clinical manifestations and follow-up. The general characteristics, clinical manifestations, the pathological and immunohistochemical characteristics, treatment options, and prognostication of the nine SCCB cases were analyzed. Statistical analysis was performed using commercially available software. Kaplan-Meier survival curves were calculated. This study was approved by the Ethics Committee of Tongji Hospital, Tongji Medical College, Huazhong University of Science and Technology.

### Literature review

In order to gain a better understanding of the clinical features of SCCB, another 119 cases reported in 56 articles (from January 1979 to March 2014) were reviewed together with the 9 cases in Tongji Hospital, Huazhong University of Science and Technology. A retrospective analysis of all the 128 SCCB cases was performed. The pathological stage, immunohistochemical markers, and survival curves of all the 128 cases were also retrospectively analyzed. Kaplan-Meier survival curves were calculated.

## Results

### Clinicopathological features

We investigated the 9 cases including 7 males and 2 females. Their ages were between 43 and 68 years old and 56 in averages. Lesions were found in the lateral wall of bladder in 7 cases, posterior wall in 2 cases, including 1 case of multiple. The sizes of tumor ranged between 1.5 cm × 2.0 cm and 6.2 cm × 5.0 cm. Intermittent painless gross hematuria were presented in all the patients and accompanied bywaist pain in 2 cases. Five male cases had smoking history. Physical examanation found that bilateral renal percussive pain is positive in 2 cases, left renal percussive pain is positive in 1 case, and deep tenderness of bladder in 1 case. All cases were scanned by ultrasound or CT. Enlargement of lymph nodes were found in 6 cases, seminal vesicle invasion in 2 cases, and rectum or uterus invasion in 3 cases. First time pathology or biopsy found transitional cell carcinoma in 5 cases, 1 case of highly differentiated adenocarcinoma, 1 case of glandular cystitis, and 1 case of inverted papilloma (Table 1).

**Table 1:** Characters of SCCB in 9 cases in Tongji Hospital

Case	Sex	Age	Size of tumor (cm)	Sites	Syndromes	Imaging features	Pathology, stage
1	M	66	6.2	Left anterior wall	intermittent hematuria, renal percussive pain	Cauliflower, wide base, left hydronephrosis, retropubic unclear boundaries	Small cell cancer, T4aN2M1
2	M	68	3.0	Posterior wall	intermittent hematuria, deep tenderness of bladder	Cauliflower, wide base, unclear boundaries of rectum	Transitional cell with small cell carcinoma T3bN0M0
3	M	43	6.0	Left posterior wall	intermittent hematuria, left waist pain	wide base, bilateral uronephrosis, seminal vesicle invasion, lymph node enlargement	Mixed carcinoma (small cell based combined with transitional cell carcinoma and adenocarcinoma) T4aN2M0
4	M	67	2.0	Left anterior wall	intermittent hematuria	Papillary, exogenous, calcification	Small cell carcinoma T3bN0M0
5	M	64	2.0	Left side wall	Hematuria, pain of bladder	Cauliflower, wide base, lymph node enlargement	Small cell cancer T2bN0M0
6	F	54	4.0	Right side wall	Hematuria, lower limbs edema	Cauliflower, lymph node enlargement	Small cell cancer with lymph node metastasis T4N1M0
7	M	67	3.0	Posterior wall	Intermittent painless hematuria,	Cauliflower, wide base,	Mixed carcinoma (small cell based combined with transitional cell carcinoma and adenocarcinoma) T2bN0M0
8	M	62	4.5	Right side and top wall	Painless hematuria, frequency, urgency	Cauliflower, multiple, boundless, lymph node enlargement	neuroendocrine small cell carcinoma with right obturator lymph node metastasis T3N1M0
9	F	70	2.5	Right side wall	Hematuria, frequency, urgency, odynuria	fumigating, base broad, gray white	Small cell carcinoma combined with some poorly differentiated transitional cell carcinoma T2N0M0

**Fig. 1:**
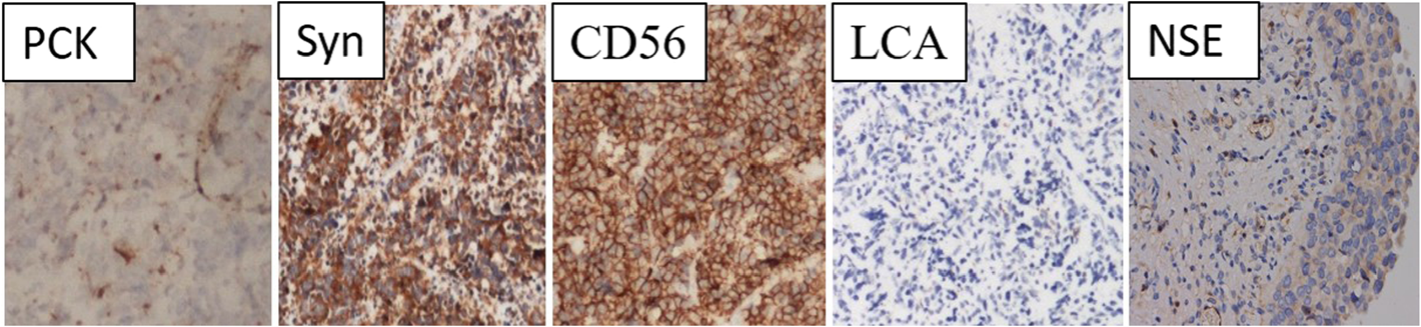
Immunohistochemical staining shows expression of different cell markers

**Fig. 2:**
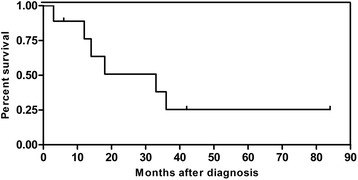
Kaplan-Meier survival curves for 9 cases

**Fig. 3:**
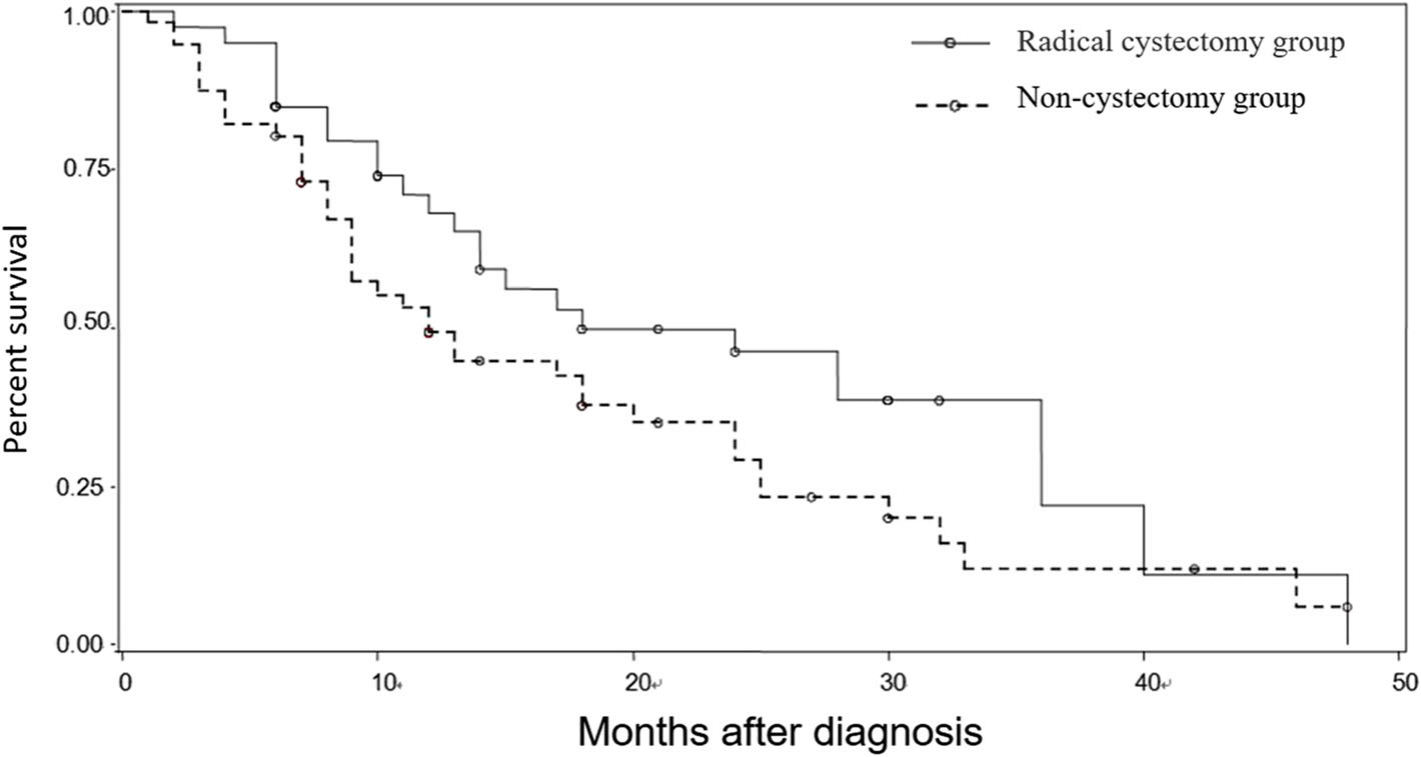
Kaplan-Meier survival curves of outcomes of difference surgeries for the 128 SCCB patients. The radical cystectomy group survival rates of 1, 2, 3 years are 68.11, 46.31, 22.05%, respectively. The non-cystectomy group survival rates of 1, 2, 3 years are 49.36, 29.24, 8.03%, respectively. The mean survival time of the two groups was 23.86 and 17.77 months, respectively. There was no statistically difference between the two groups (Log-rank test, *X* ^2^ = 2.6041, *P* = 0.1066)

After definite diagnosis, 9 cases were treated with corresponding surgery according to different conditions and stages of tumors (Table 2). Postoperative pathology showed different histopathological conclusions (Table 3) (Fig. 1). When recovered from operation, 4 cases were treated with chemotherapy and only 1 case was with radiotherapy. Four cases received partial cystectomy. After at least 6-month follow-up, Kaplan-Meier survival curves for 9 cases showed survival rates (Fig. 2). According to published reports in China, we analyzed outcomes of different surgeries for the 128 SCCB patients (Fig. 3).

**Table 2:** Histopathological feature of SCCB in 9 cases

	NSE	PCK	Syn	CD56	CgA	LCA	EMA	Ki-67Li
1	+	+	+	+	+	−	+	−
2	+	+	+	+	−	−	+	−
3	+	+	+	+	−	−	−	−
4	+	+−	+	+	−	−	+	99%
5	+	+	+−	+	−	−	+	−
6	+	+	+	+	+	−	−	−
7	+	+−	+	+	+	−	−	60%
8	+	+	+	+	+	+	−	90%
9	+	+	+−	+	+	−	+	60%

*NSE* neuron specific enolase, *PCK* broad-spectrum cytokines, *Syn* synaptophysin, *CgA* addicted chromogranin A, *LCA* leukocyte common antigen, *EMA* epithelial membrane antigen, *Ki67* status labeled antigen

**Table 3:** Treatment characteristics and follow-up

	1	2	3	4	5	6	7	8	9
PRB		√		√			√		√
TRB			√		√	√		√	
LND			√		√	√		√	
RBI			√		√	√		√	
SCS	√								
BI		√		√			√		√
CT	√				√	√		√	
RT			√						
TTL	3 M	12 M	14 M	>6 M	>40 M	>18 M	>84 M	36 M	33 M
PG	Systemic metastasis	Myocardial infarction	Systemic metastasis	No recurrence	Survive without tumor	Systemic metastasis	Survive without tumor	Ascites	Cachexy

*PRB* partial resection of bladder, *TRB* total resection of bladder, *LND* Lymph node dissection, *RBI* reconstruction of bladder with intestinal tract, *CS* skin colostomy surgery, *BI* bladder irrigation, *CT* chemotherapy, *RT* radiotherapy, *TTL* time to live, *PG* prognosis

**Fig. 4:**
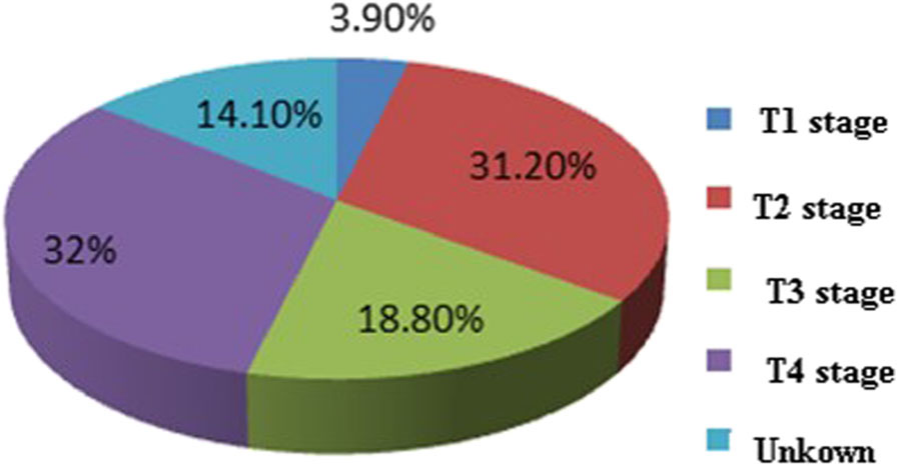
Pathological stage ratio of 128 cases

**Fig. 5:**
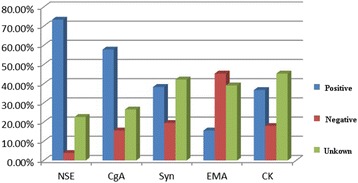
Features of immunohistochemical markers of SCCB in 128 cases

**Fig. 6:**
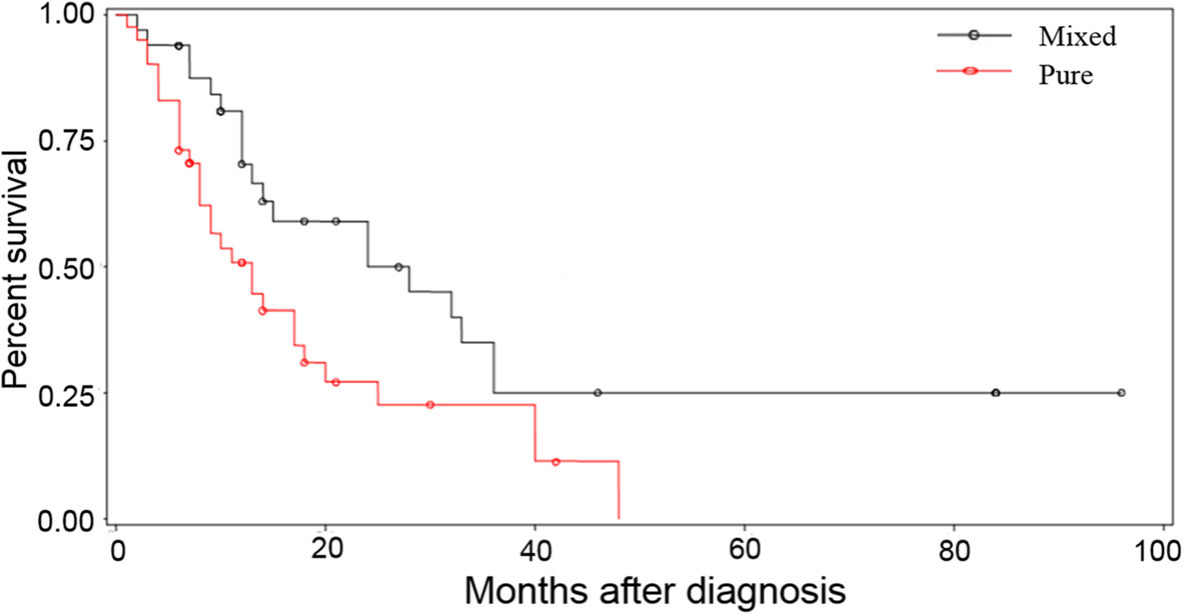
Kaplan-Meier survival curves of outcomes among pure small cell type and mixed cell type in the 128 SCCB patients. The mixed cell type group survival rates of 1, 2, 3 years are 70.4, 50.0, 25.0%, respectively. The pure small cell type group survival rates of 1, 2, 3 years are 47.5, 24.2, 13.6%, respectively. The mean survival time of the two groups was 23.5 and 18 months, respectively. There was statistically difference between the two groups (Log-rank test, *X* ^2^ = 5.547, *P* = 0.019)

We also analyzed pathological types of 128 patients, in which 51/128 (39.8%) were isolated small cell carcinoma, and 72/128 (56.3%) were mixed small cell carcinoma. In all cases, tumors in 53/72 cases were combined with transitional cell carcinoma (73.6%), 8/72 with squamous cell carcinomas (11.1%), 5/72 with adenocarcinoma (6.9%), 4/72 with carcinoma in situ (5.6%), 3/72 with three kinds of cells composition (4.2%) (Fig. 4). Even in 2 cases, the tumors were composited of four kinds of cells including transitional cell carcinoma of bladder, small cell carcinoma, squamous carcinoma, and adenocarcinoma. SCCB is featured with various epithelial or nerve secretory symbols. One hundred and three out of 128 cases express one or more neuroendocrine markers such as NSE, CgA, and Syn. NSE is the most sensitive marker and its positive rate is 73.4%. EMA has the highest positive rate among epithelial symbols (62.5%) and followed by keratin (61%), CEA (50%), and Leu-M1 (43.8%) (Fig. 5). Kaplan-Meier survival curves showed the outcomes between pure small cell type and mixed cell type of SCCB are different (Fig. 6).

**Fig. 7:**
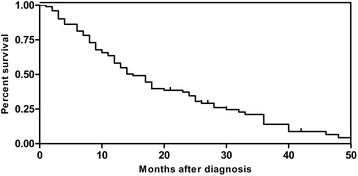
Kaplan-Meier survival curves of 128 SCCB after diagnosis

Many studies suggest that NSE, CgA, Syn, and etc. play important roles in SCCB diagnosis. Epithelial membrane antigen has no specificity, but important in differential diagnosis. For example, LCA is positive in lymphoma but not expressed in SCCB. Despite of a variety of treatments, SCCB has poor overall prognosis for fast growth, strong invasion, and early metastasis (Fig. 7).

## Discussion

We analyzed retrospectively nine cases with SCCB diagnosed between 1980 and 2014. Combined with published reports, we investigated clinical symptoms, histopathological characters, treatments, and prognosis to enhance the understanding of SCCB.

Incidence of bladder cancer accounted for 5% of malignant tumor, and it is the second most common urologic malignancy. Up to 95% bladder cancer are of epithelial origin, among which 90% are urothelial neoplasms and followed by squamous cell carcinoma (5–8%) and adenocarcinoma (1–2%) [3]. Non-epithelial tumors are rare, such as small cell carcinoma and sarcoma. SCCB is a highly malignant tumor that occurs mainly in lung and esophagus. Extra-pulmonary small cell carcinoma accounted for 4% of all small cell carcinoma [4]. The disease was firstly described in 1981 by Cramer, and its incidence accounted for 0.5–1% of primary bladder tumors. About 50% SCCB are compound bladder cancers that combined with other histological bladder tumors. Average age of incidence is about 66.9 years old and 79% patients have a history of smoking. Males have three to five times incidence higher than female. So smoking may be one of the causes of the illness. Other environmental and individual risk factors are not confirmed as the etiology, but the underlying factors include chronic cystitis, bladder stones and cystoplasty [5]. Up to now, pathogenesis of SCCB is not clear. Similar to lung small cell cancer, SCCB may be associated with chromosomal aberrations, such as certain sites lacking tumor suppressor genes or chromosomes areas containing cancer-causing genes (such as c-MYC) abnormal amplification. Silence of the tumor or metastasis suppressor gene is considered to be another potential mechanism of bladder cancer [6].

Histopathological forms of SCCB have no obvious difference with general bladder tumors. It belongs to a part of neuroendocrine tumor group. Characteristically, small amounts of neurosecretory granules in 150 ~ 250 nm diameter can be found under the electron microscope. About 50% SCCB contains the other kinds of cancer, such as transitional cell carcinoma, adenocarcinoma, and squamous carcinoma. It can also be associated with transitional epithelial atypical hyperplasia or carcinoma in situ. The possible reason is that SCCB is derived from the neural crest of submucosa all-around poorly differentiated stem cell population [7]. SCCB diagnosis mainly depends on cystoscopic biopsy lesions and postoperative histopathological examination, such as measurements by light microscopy cell morphology, cell ultrastructure electron microscope examination, and immunohistochemical examination. Preoperative examinations also include imaging, such as CT or MRI scan, urine cytology, cystoscopy, and biopsy. Cytology of SCCB is similar with poorly differentiated squamous cell carcinoma, adenocarcinoma, and transitional cell carcinoma, so diagnostic identification relies on electron microscopy or immunohistochemistry [8]. Eight cases in our report were mistaken as transitional cell carcinoma, inverted papilloma, and glandular cystitis et al. before diagnosed. Since there are clear distinctions in treatment programs between SCCB and other bladder tumors, we should raise awareness of the disease and carefully check the tissue samples in order to avoid misdiagnosis, missed diagnosis, and delay of treatment ﻿timing.

Since SCCB is clinically rare tumor, there is no standard treatment. In absence of multi-center randomized study, most treatments are multi-mode combination therapy based on single-center retrospective study, so that its variability in treatment programs is great. Most scholars believe that SCCB is a mixed cell carcinoma and advocate the preferred surgical treatment, including radical cystectomy, partial cystectomy, or transurethral resection of bladder tumors (TURBT). Parts of scholars believe that the majority of SCCB has occurred micro-metastases locally at the time of diagnosis. ﻿Therefore﻿, surgery alone is not enough and the combination of radiotherapy and chemotherapy is needed [9]. In our analyzed group, 102 patients were treated with surgery in 128 cases (79.7%), in which TURBT, partial resection and radical resection account for 41/102 (40.2%), 15/102 (14.7%), and 46/102 (45.1%), respectively. We recommend surgery as the priority. Chemotherapy plays a prominent role in the management of SCCB. Bladder cancer cells are sensitive to chemotherapy, especially transitional cell cancer, but partial bladder infusion chemotherapy cannot effectively prevent cancer metastasis. Systemic chemotherapy based on platinum was previously advocated for SCCB treatment. Mackey retrospectively analyzed 106 cases of SCCB. He believed that cisplatin-based chemotherapy was an independent prognostic factor and should be used in the treatment [10]. Choong thought that chemotherapy should be used on III and IV periods and not recommended for II period [11]. Abbas analyzed retrospectively that the average survival time of radical cystectomy surgery combined with chemotherapy was 21 months [12]. Siefker reported that neoadjuvant chemotherapy could reduce preoperative staging and was beneficial for surgical removal to prolong survival time of patients [13]. Etoposide, cisplatin, and carboplatin are usually used for pure SCCB and methotrexate, vincristine, adriamycin, cyclophosphamide, etoposide, or cisplatin for SCCB combined with transitional cell carcinoma. In our study, chemotherapy (local + systemic) and radiation therapy respectively﻿﻿ account for 48.5% and 34.6% of 128 cases. Lack of unified chemotherapy treatments, chemotherapy drugs cannot be comparable. For SCCB with skull or bone metastasis, radiation therapy may be recommended. In addition, local chemotherapy via internal iliac catheter can be applied for patients with poor physical fitness or unresectable primary tumors.

Distant metastasis occurs in 78% of patients. The patients with treatments had a better median survival of 12 to 24 months compared to the patients without treatments of 4 to 5 months. Trials reported that 2-, 3-, and 5-year survival rates of SCCB were 20, 13, and 8%, respectively [14]. Choong et al. studied retrospectively that 1-, 3-, and 5-year survival rates of 44 cases of SCCB were 61.4, 27.3, and 25%. In our study, the average survival time was 20.54 months; 1-, 2-, 3-, and 4-year survival rates were 57.78, 36.94, 16.61, and 2.97%, respectively.

## Conclusions

SCCB is a malignant tumor with lower degree differentiation and a highly invasion and metastasis. It is clinically rare. Majority of patients with SCCB have hematuria symptoms. When diagnosed, it is often already advanced so that the prognosis is poor. SCCB has unique biological characteristics with positive neuroendocrine markers. The most sensitive marker is NSE that has well specification. Hence, diagnosis of SCCB depends on pathology and immunohistochemistry. Radical bladder cystectomy combined with chemotherapy or radiotherapy can improve survival time. The outcomes of different cell types of SCCB are different. Surgery is still the main treatment of SCCB, but multi-mode integrated treatment should be a better alternative. Treatment schedules of SCCB refer to the current small cell lung cancer principles. It is difficult to determine the best options through prospective studies. Early detection, diagnosis, and treatment are the key to improve or prolong the survival time of patients.
